# Health effects of European colonization: An investigation of skeletal remains from 19th to early 20th century migrant settlers in South Australia

**DOI:** 10.1371/journal.pone.0265878

**Published:** 2022-04-06

**Authors:** Angela Gurr, Jaliya Kumaratilake, Alan Henry Brook, Stella Ioannou, F. Donald Pate, Maciej Henneberg

**Affiliations:** 1 Biological Anthropology and Comparative Anatomy Research Unit, Adelaide Medical School, University of Adelaide, Adelaide, South Australia, Australia; 2 Discipline of Anatomy and Pathology, Adelaide Medical School, University of Adelaide, Adelaide, South Australia, Australia; 3 School of Dentistry, University of Adelaide, Adelaide, South Australia, Australia; 4 Institute of Dentistry, Queen Mary, University of London, London, United Kingdom; 5 Archaeology, Flinders University, Adelaide, South Australia, Australia; 6 Institute of Evolutionary Medicine, University of Zurich, Zurich, Switzerland; Appalachian State University, UNITED STATES

## Abstract

The British colony of South Australia, established in 1836, offered a fresh start to migrants hoping for a better life. A cohort of settlers buried in a section of St Mary’s Anglican Church Cemetery (1847–1927) allocated for government funded burials was investigated to determine their health, with a focus on skeletal manifestations associated with metabolic deficiencies. Findings of St Mary’s sample were compared with those published for contemporary skeletal samples from two British cemeteries, St Martin’s, Birmingham, and St Peter’s, Wolverhampton, to explore similarities and differences. To investigate the changing economic background of the St Mary’s cohort, which may have influenced the location of their burial within the cemetery, the number and demographic profile of government funded burials and those in privately funded leased plots were compared. The study sample consisted of the skeletal remains of 65 individuals (20 adults, 45 subadults) from St Mary’s Cemetery ‘free ground’ section. The bones and teeth of individuals in this cohort showed evidence of pathological manifestations, including areas of abnormal porosity in bone cortices in 9 adults and 12 subadults and flaring of metaphyses (one subadult) and costochondral junctions of the ribs (one subadult). Porous lesions of orbital roof bones (Types 3 to 4) were seen on three subadults. Macroscopic examination of teeth identified enamel hypoplastic defects and micro-CT scans showed areas of interglobular dentine. Comparison of St Mary’s findings with the British samples revealed that prevalences of manifestations associated with vitamin C deficiency were higher at St Mary’s and manifestations associated with vitamin D deficiency were lower respectively. The location of burial pattern at St Mary’s Cemetery, from the mid-1840s to1860s, showed differences in the economic status of migrants. This pattern changed from the 1870s, which reflected improvements in the local economy and the economic recovery of the colony.

## Introduction

Early 19^th^ century migrant settlers in the new British colony of South Australia would have hoped for a better life than they had experienced in Britain. The palaeopathological investigation of this rare skeletal sample from the ‘free ground’ area of St Mary’s Anglican Church Cemetery, near the city of Adelaide, South Australia, allows an insight into some of the health and economic challenges that these migrant settlers faced. The free ground section of this cemetery was allocated for individuals whose burials were paid for by the Government of South Australia, when they or their families did not have the funds to cover the costs. This study investigates the health status of this small cohort of settlers who lived in the region of the village of St Marys-on-the-Sturt from the 1840s to the1920s, with a focus on the pathological manifestations that may indicate a disturbance of the metabolism. To determine if the health of these individuals was different from their British contemporaries, findings are compared to published data for skeletal samples from St Martin’s Cemetery in Birmingham, and St Peter’s in Wolverhampton. Data from St Mary’s Church records and headstones associated with privately funded burials in leased plots in this cemetery, provide information to compare the number of burials in each section of the cemetery, as well as assemble demographic profiles including seasonality of death. These data are valuable in gaining an understanding of the lives and deaths of these settlers and any changes that occurred in burial location patterns within the cemetery (1847–1927), to provide the economic background for the cohort, for example, was the free ground area of the cemetery used continuously during the study period or was there a ‘peak’ in the burial numbers? How many infants under one year of age were buried in the free ground?

The aims of this study are to:

1. Investigate skeletal remains of a group of migrant settlers to South Australia who were buried in the free ground area of St Mary’s Anglican Church Cemetery from 1847 to 1927, with a focus on the pathological manifestations that may indicate a disturbance of the metabolism.
2. Compare the findings of the St Mary’s samples with those published for individuals buried at two 19^th^ century British cemeteries to explore similarities and differences in health, particularly skeletal manifestations associated with metabolic deficiencies.
3. Compare the number, percentage and demographic profiles of the cohorts buried in the government funded free ground area of St Mary’s Cemetery and those in privately funded leased burial plots during the study period (1847 to 1927), to investigate if there were any changes in the pattern of burial locations within St Mary’s Cemetery.

### A new non-custodial colony

Industrialisation of Britain during the 19^th^ century altered the landscape of many parts of the country, both urban and rural, the economy and the lives of many people. The rapid expansion of British industries led to the marked increase in employment opportunities and this in turn led to the mass migration of workers to industrialised centres. Most of these centres lacked the infrastructure to cope with the increase in the population size. Many people had to live in overcrowded buildings, where the sanitary conditions were poor and water supplies could be easily contaminated [1–3]. These conditions, together with long hours of working inside factories powered by coal fired engines and diets lacking in essential nutrients, contributed to the poor health of the working classes [2–7].

The indutrialisation of many urban centres also affected the traditional smaller industries in rural regions of Britain. The inability to compete with the low production costs of commercial items in large factories led to the closure of many small rural trades [8–10]. Furthermore, importation of large quantities of raw materials, such as iron, copper and tin ores at cheaper prices to feed large industries caused the downturn of regional mining industries [9, 10]. Poor harvests and the spread of disease in potato crops (blight), further affected farmers [8–11]. The net outcome was unemployment and economic hardships in regional and rural areas of Britain.

The British government encouraged the migration of people to new colonies in order to reduce overcrowding in industrialised centres and unemployment in regional and rural parts of the country. South Australia was one such colony and established partly to help solve some of these problems [12, 13]. The South Australian Act (1834) allowed the sale of land in the proposed new settlement to individuals who would establish primary industries such as farming, mining and manufacturing [13–15]. Thus, emigration to South Australia was extensively advertised in Britain [16]. The vast size, climate and the natural environment of South Australia for farming opportunities may have attracted many people to migrate [16]. Development of this new Australian colony required builders, mechanics, agricultural labourers and miners who wished to create their own opportunities [17]. A high number of migrants to South Australia came from the counties of Cornwall, Devon, Dorset and Somerset, followed by Lancashire, Middlesex, Staffordshire and Warwickshire [16, 18, 19].

The policy for migration to South Australia was designed to maintain a regular supply of skilled workers to landowners, new industries and for the continued development of the infrastructure and new settlements [16, 17, 20]. Migration was regulated by the Colonial Land and Emigration Commission (CLEC), whose agents selected healthy young males and females of good character in equal numbers [16, 17, 20]. Skilled migrants who could not afford the cost of the voyage to South Australia were encouraged to apply for an assisted passage. The assisted passage program was funded by the British Government and the South Australian Company. Individuals selected for an assisted passage had the opportunity to obtain additional financial support from local charitable organisations. This money covered the cost of transport to the port of departure and/or the compulsory deposit required for bedding, utensils and a set of clothing for all weathers during the long voyage [16, 20].

In total, 186,054 indivduals migrated to South Australia between 1836–1900 from Britain. The government assisted passage was received by 123,039 migrants (66% of total) [17]. The CLEC selection criteria were not applied to individuals who paid the full costs of their passage and had adequate funds to support themselves in the colony. The health of migrants, who travelled to South Australia was considered a high priority by the British Government. Therefore, the CLEC monitored conditions on board by appointing a qualified surgeon superintendent on each ship, who was accountable for the health and wellbeing of all passengers [16, 20, 21].

Political disagreements within the new South Australian Government delayed the surveying of land for migrant settlements and the development of supporting infrastructure [12]. This affected the initial establishment of farms, food production, industrial enterprises and permanent housing for migrants. These issues also delayed the development of the economy, caused unemployment and led to the removal of the first Governor, Captain John Hindmarsh. The second Governor, Lieutenant Colonel George Gawler, was appointed by the British Government in 1838 [19, 22]. This Governor had a proactive approach and commissioned multiple infrastructure projects to rapidly develop the colony [22, 23]. However, the cost of these projects was very high, and the British government refused to pay the expenditure and recalled Governor Gawler back to London in 1841 [19, 24].

South Australia’s third Governor in five years, Captain George Grey, was appointed in 1841. During this decade the colony faced its first economic depression [19, 25]. A lack of funds to continue with public infrastructure developments and other works meant Governor Grey faced retrenchment from1841 to 1845. His decision to redirect the majority of the unemployed workforce into agricultural industries [12, 19], resulted in an increase of agricultural goods, particularly wheat and animal products. This in turn led to the establishment of export industries to other Australian colonies and Britain [12, 15].

### Destitute in South Australia

The climatic conditions of South Australia, such as high summer temperatures and limited rainfall, often resulted in periods of drought and poor harvests. These conditions may have also contributed to the poor economic growth and the unemployment experienced during the development of the colony. Throughout this period, life for many settlers was harsh, particularly those who were unemployed and had to depend on charitable organisations and/or the government for their survival [23]. State Records of the Government of South Australia [26], state that 446 sick and destitute people received help in the form of food rations from the Emigration Department in 1839–1840. This number increased to 904 persons during the period of 1840–1841 [23]. The Maintainace Act of 1843 [27], was passed to address the care of “deserted wives and children and other destitute persons” [27:1]. The establishment of the Destitute Board followed six years later in 1849. This board offered support to the elderly, chronically infirm and some widows. The Board also initiated the construction of the Destitute Asylum in the city of Adelaide during the 1850s [23, 28, 29].

The Destitute Asylum was modelled on the British workhouse system [28–30], with strict regulations, such as complusory wearing of uniforms for inmates and severe penalties if regulations were not followed [23, 30]. Admittance to the Destitute Asylum to receive indoor “relief” was a last resort and individuals had to prove that they had no other relatives or means of support [27, 30]. Deserted women with children could apply for admission to the asylum [30] and expectant mothers considered to be destitute could also stay for up to six months [23, 30]. Individuals, who still had their own accommodation but no other support were given weekly food rations and firewood from the Asylum as outdoor “relief” [30]. People who lived in rural areas either had to travel long distances into the city of Adelaide to receive support from the asylum or cope as well as they could within their own community.

### Health in the early colony

Potential health burdens that early settlers may have faced include the spread of infectious diseases such as diphtheria, typhoid, typhus fever and tuberculosis, and/or metabolic deficiencies resulting from hardships faced in their new environment [31–37]. Individuals who could not afford the cost of local medical services may have been badly affected, as the only public hospital was located in the city. This meant people living in rural areas may have had limited access to health services or had to travel long distances to receive treatment [36, 37]. Some of the above mentioned diseases, as well as the interactions between them, may in some chronic cases have caused changes in the bone or tooth morphology [38–42]. An example of morphological changes in bone can be seen with a chronic deficiency of vitamin D, which can cause pathological manifestations in bones of the skeleton such as bending distortion in long bones [38]. A number of skeletal manifestations, such as abnormal porosity of cortical bones, enlargement and flaring of costochondral junctions of ribs and/or porous lesions on the bones of the orbital roof, have been previously interpreted as signs of chronic metabolic disturbance or deficiencies [43–47].

Bones of the skeleton are a dynamic tissue and undergo remodelling during life, in response to varying forces acting upon them [48]. Therefore, disease manifestations seen in skeletal remains, particularly in bone cortices, had occurred during the last remodelling that took place before death. Careful investigation of the characteristics of the manifestation/s and the pattern of distribution overall among the bones of the body may help to identify metabolic deficiencies that a person had experienced [43, 44, 49].

Abnormal porous lesions on bone cortices result from defective calcification of the bone matrix [50, 51]. Vitamin C and vitamin D deficiencies affect collagen synthesis [52–54] and affect mineralisation [44, 55, 56], respectively. Both processes could produce abnormal porosity of the bone cortices seen in archaeological skeletal samples. Determining the aetiologies of porous lesions on the bones of the orbital roof (often referred to as cribra orbitalia) has been controversial [49, 57, 58]. The morphology of these porous lesions can vary according to the processes that occurred to produce them. These may include subperiosteal inflammation in relation to a deficiency of vitamin C, vitamin D, vitamin B12, and/ or related to infection [43, 44, 47, 59, 60]. Some porous lesions in this anatomical location have been associated with anaemia. When haemoglobin becomes inadequate, due to the lack of iron in the body, the red marrow compensates by the overproduction of red blood cells and proliferates causing the expansion of the trabecular bone and of the diploe [61–63]. In some chronic cases of anaemia, the surface cortical bone of the orbital roof may ‘thin out’ and expose the underlying trabecular bone, which could appear as porous lesions. Iron deficiency anaemia could result from chronic bleeding, malabsorption of iron in the gut and/or parasitic infections and/or a dietary deficiency of iron [59, 62–66]. Furthermore, hemoglobinopathies such as hereditary anaemia, a combination of the above conditions, or other aetiologies should also be considered [63, 67].

Enamel is a highly specialised dental tissue, in which the only responses to various insults during tooth development are to form hypoplastic or hypomineralised enamel or both [41, 42, 68]. Unlike bone, it does not remodel after the development of the tooth is complete [40, 41, 68]. Therefore, any lesion/s produced by pathological processes that disrupt the enamel producing ameloblast cells remains in the tooth for the rest of an individual’s life. Tooth development commences at six weeks in utero and continues until approximately 21 to 23 years of age [69, 70]. After eruption, the tooth can be affected by erosion, abrasion, attrition and caries, which may over time affect the appearance of enamel defects [68].

Dentine is also a highly sensitive tissue which can be affected by health insults during the development of the dentition. Such insults could disrupt the mineralisation process of the dentine and produce areas of under-mineralised matrix referred to as interglobular dentine (IGD) [41, 42, 68]. Unlike enamel, dentine is a vital tissue due to the dentine-pulp complex that allows dentine to respond to caries (decay), erosion, and trauma after the development of the tooth is complete [41, 42, 68]. However, the remodelling process in dentine is much less than that in bone [42]. Therefore, teeth are a valuable source of information for the investigation of the effects of the environment on an individual’s health.

### St Marys-on-the-Sturt—A rural settlement in the colony

**Fig 1:**
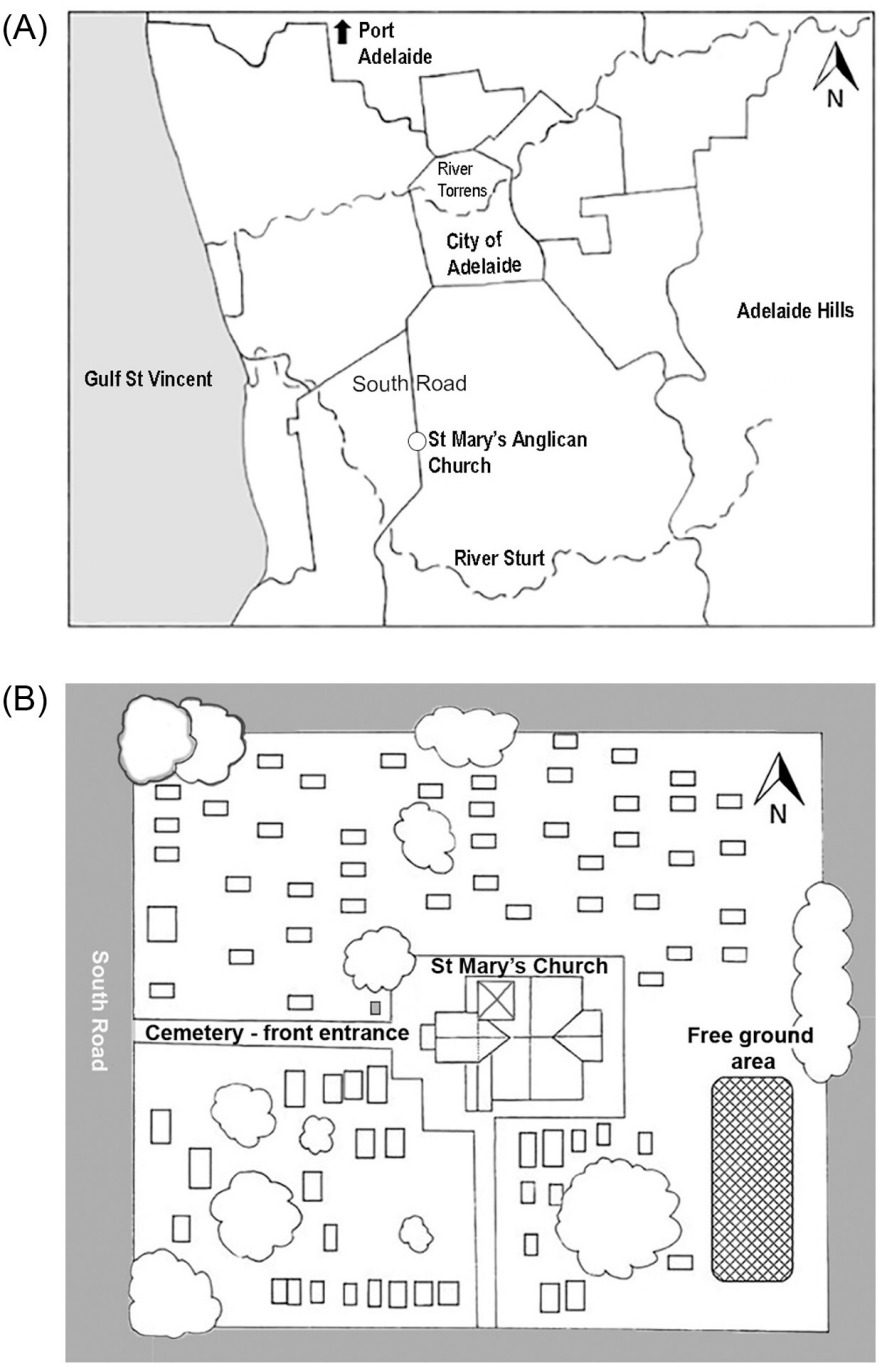
**A**. Location map: Position of St Mary’s Church and Cemetery in relation to the City of Adelaide and surrounding region. Reprinted [75] and under a CC BY license, with permission from the City of Marion Council, original copyright 2020. **B**. A schematic diagram of St Mary’s Anglican Church Cemetery. The hatched rectangle at the rear of the church building shows the free ground area. Illustrative purposes only and not to scale or representative of the number of burials/gravestone memorials in this cemetery.

St Marys-on-the-Sturt was established in the late 1830s [71]. This was a small village located eight kilometres south of the city of Adelaide (Fig 1A). The majority of migrants were of British origin and an Anglican Church (St Mary’s) was established in the village. The land surrounding the church was allocated for a cemetery (Fig 1B) and the first burial was in 1847 [72]. A small section of the cemetery at the rear of the church was allocated for individuals whose burials were funded by the Government of South Australia [73, 74]. This section was referred to as the ‘free ground’ area and the burials here were unmarked (Fig 1B) [71].

## Material and methods

### Sample

The Flinders University Social and Behavioural Research Ethics Committee (SBREC project number 8169) approved the research. The excavation and the study of skeletal remains were conducted at the request of the St Mary’s Anglican Parish. The burials at the free ground area were unmarked, thus preventing the identification of the buried individuals. No permits were required for the described study, which complied with all relevant regulations [73].

Skeletal remains of 70 individuals (20 adults and 50 subadults) were excavated in 2000 [73], from the free ground section of St Mary’s Anglican Church Cemetery, South Road, South Australia. The individuals had been buried in this area of the cemetery between 1847 and 1927. Skeletons were not excavated from any other areas of the cemetery. A site code (SMB—St Mary’s Burial) and identification number were allocated to each excavated skeleton [73]. Skeletal remains of St Mary’s sample are part of an archaeological collection; thus, no destructive analysis was permitted.

Previous investigations: Immediately after the excavation (in 2000), an examination of the skeletal samples was conducted by Timothy Anson [73]. This included an assessment of i) the state of preservation of the remains, ii) an estimation of age range at death and iii) determination of sex. A summary of the methods used are given below but full details of the methods, systems, and categories used for these estimations can be found in Anson [73].

i) The state of preservation of each skeleton was estimated as very poor, fair, good to very good [73].
ii) Age range at death:
  Subadults: Dental development and eruption rate charts were used in conjunction with assessment of changes in ossifiction centres of the skeleton during development. [76–78].
  Adults: Multiple skeletal changes were investigated such as morphological changes in the pubic symphysis and auricular surface, and stature [76, 79–81]. Degenerative changes in the dentition were also used as an indicator of age-related changes [82]. The accuracy of this method is subjective, and results may differ in each individual [83].
iii) Estimation of sex
  Subadults: Determination of sex was not possible for the subadults from St Mary’s sample due to a lack of changes associated with sexual maturity.
  Adults: Observations of morphological changes described by Buikstra and Ubelaker [76], Scheurer et al. [78] and Bass [84] were used to attribute sex.

The St Mary’s skeletal collection is temporarily held in an osteological laboratory at the University of Adelaide, South Australia. The unidentified specimens are the property of St Mary’s Anglican Church, with Flinders University, South Australia, having a professional oversight of the collection.

### Scoring of skeletal material

Current study: Examination of the St Mary’s samples found that the skeletons of five subadults were in a very poor state of preservation (i.e., extremely fragmented with little cortical bone available). Therefore, these subadults were excluded from this investigation, thus the remaining total sample size was of 20 adults and 45 subadults. Skulls from eight subadults had disintegrated post-mortem and therefore were not examined.

### Macroscopic examination

Each skeleton was arranged in the anatomical position and an inventory of the bones was prepared as described by Buikstra and Ubelaker. [76] and Mitchell and Brickley [85].

The anatomical sites and criteria used for the identification of skeletal manifestations associated with metabolic deficiencies are extensive and are presented in S1 Table. Criteria used for this assessment were as described by Ortner et al. [43], Brickley et al. [44], Brickley and Ives [45], Ortner and Mays [46], Ortner & Ericksen [47], and Heron and Grauer [86] and the characterisation of abnormal porosity was taken from Ortner et al. [43], Brickley et al. [44] and Ortner & Ericksen [47].

Porous lesions seen on the bones of the orbital roof were scored according to the method described by Stuart Macadam [59:109], for example, Type 1- “capillary-like impression on bone”, Type 2- “scattered fine foramina”, Type 3 to 5 -ranged from “large and small isolated foramina to outgrowths from trabecular bone that extended to the surface of the outer table.

Enamel hypoplastic defects were recorded using an adaptation of the Enamel Defect Index (EDI), as described by Brook [87], Brook et al. [88], and Elcock et al [89]. The above investigation was carried out using a magnification lamp.

### Micro-CT examination

Investigation of the internal structure of tooth samples for interglobular dentine (IGD) was part of the study. Traditionally, histological techniques were used for this type of investigation, which required sectioning of the tooth sample. Consequently, a non-destructive method, X-Ray Computed Tomography (micro-CT) was used. The cost of this method allowed the investigation of only a selected sample of teeth from the St Mary’s skeletal collection. One tooth from each of the 19 individuals was selected. The individuals were from a broad age range (~2 years to 60+ years of age). The collected tooth samples included two permanent incisors, three permanent canines, three permanent premolars, nine permanent first molars, and two primary molars, as the same tooth type was not available from each individual. Each tooth was scanned using the Bruker SkyScan 1276 Micro-CT scanner at Adelaide Microscopy, The University of Adelaide [90]. The scanner was set at source voltage: 100 kV, source current 200 μA, camera binning: 4032 x 2688, filter: aluminium and copper, and pixel size: 9.0 μm. The tooth sample from SMB 63 was scanned for a second time using the pixel size of 5.21 μm. Micro-CT scan datasets were reconstructed into a visual image using NRecon, a volumetric reconstruction software. These reconstructed scan data sets were viewed as either two-dimensional (2D) or three-dimensional (3D) images using Dataviewer, a volume rendering software and Avizo 9 software [91]. The 2D and 3D images were analysed to identify mineralisation defects in the teeth. Dentine defects seen on the micro-CT scans were scored, as described by Colombo et al. [92] and Veselka et al. [93].

### St Mary’s Cemetery burial records

Data from St Mary’s Church records, in relation to burials in the ‘free ground’ area of the cemetery (1847 to 1927) were used [73:356–381]. Parish burial records recorded the location of an individual’s burial site within St Mary’s Cemetery. Burials were either in ‘lease’ plots, which were privately funded in the main section of the cemetery (Fig 1B), with a memorial marker (i.e., headstone), or in the unmarked government funded ‘free ground’ area at the rear of the church building (Fig 1B). If an individual was buried in a ‘leased’ plot the church register recorded the assigned burial plot number and the personal details of the interred individual, plus any notes regarding the funeral arrangements (73:38). For individuals buried in the free ground section of this cemetery, minimal information was recorded. Some individuals whose names are listed in this burial register did not have their burial location site recorded. This was especially true for many infants. Some infants just had the words “unbaptised-no service” recorded (73:362). It is difficult to know if the individuals without the location of their burial recorded were interred in the free ground area of St Mary’s Cemetery. However, as the leased plots were paid for by the individual or their family, the details of the interred would have been recorded in full with the identifying number of the assigned burial plot and it is unlikely that such minimal details would have been acceptable. Therefore, for the purpose of this study individuals who did not have a location for their burial recorded in the church records were included in a list of individuals that *could* have been buried in the free ground area of St Mary’s Cemetery.

### Comparison of St Mary’s findings with those published for two British skeletal samples

Findings from the St Mary’s samples on pathological manifestations that could indicate a disturbance of the metabolism were compared with those published for the two-19th century to early 20^th^ century British skeletal samples. This was to assess the effect of the establishment of a new colony on early migrants. One sample was from St Martin’s-in-the-Bullring Church, Birmingham, England (N = 406) [45, 94, 95], where the majority of burials were between 1810 and 1864, with declining numbers of individuals interred until 1915 [94]. St Martin’s Cemetery was located in an industrial city where many individuals buried may have been from the working classes. Thus, this sample was considered appropriate for the comparison with the St Mary’s sample, as many of the buried individuals were British migrants and were from a similar socioeconomic working-class background.

The published results for the second comparison sample were individuals from St Peter’s Collegiate Church overflow burial ground, Wolverhampton, England, (1819 to approximately 1900) [96, 97]. Wolverhampton was originally a market town with a similar mix of agriculture and small industries to St Mary’s-on-the-Sturt. The industrial development of local mining activities during the 19^th^ century eventually contributed to the increase in population size in this British town [96]. Published findings from St Peter’s skeletal samples were also considered as an appropriate comparison sample to the St Mary’s sample. The majority of the individuals in St Peter’s sample were from agricultural, industrial and mining backgrounds, and thought an appropriate sample for comparison with the results of St Mary’s collection [16, 17, 96, 97].

## Results

### Demography

Estimated age range at death and the sex for the skeletal samples from St Mary’s free ground are presented in Table 1. Among the 45 subadults in this sample, 36 were under two years of age.

**Table 1:** Demography. Estimated age range and sex of St Mary’s sample [72].

Age range at death (years)	Sex			Total
	Female	Male	Undetermined	
0–1	0	0	17	17
1–4	0	0	22	22
5–9	0	0	3	3
10–14	0	0	3	3
15–19	1	0	0	1
20–29	1	0	0	1
30–39	2	1	0	3
40–49	3	2	0	5
50–59	1	7	0	8
60+	0	2	0	2
Subtotal	8	12	45	65
**Total sample**				**65**
**Adult**	8	12	0	**20**
**Subadults**	0	0	45	**45**

### St Mary’s Cemetery burial records

Data from burial records for St Mary’s Church Cemetery for 1847–1927 (73:356–381), listed individuals with the location of their burial as the free ground (Fig 1B). Some individuals did not have the location of their burial site recorded. As previously discussed, for the purpose of this study, both these types of listings (i.e., burials in free ground and no burial location recorded) in the burial records were considered to be individuals who *could* have been buried at the free ground area of St Mary’s Cemetery. The number of individuals included in this list amounted to N = 195. This number was made up of 71 individuals listed as buried in the free ground and 124 individuals with no location of burial site recorded.

**Fig 2:**
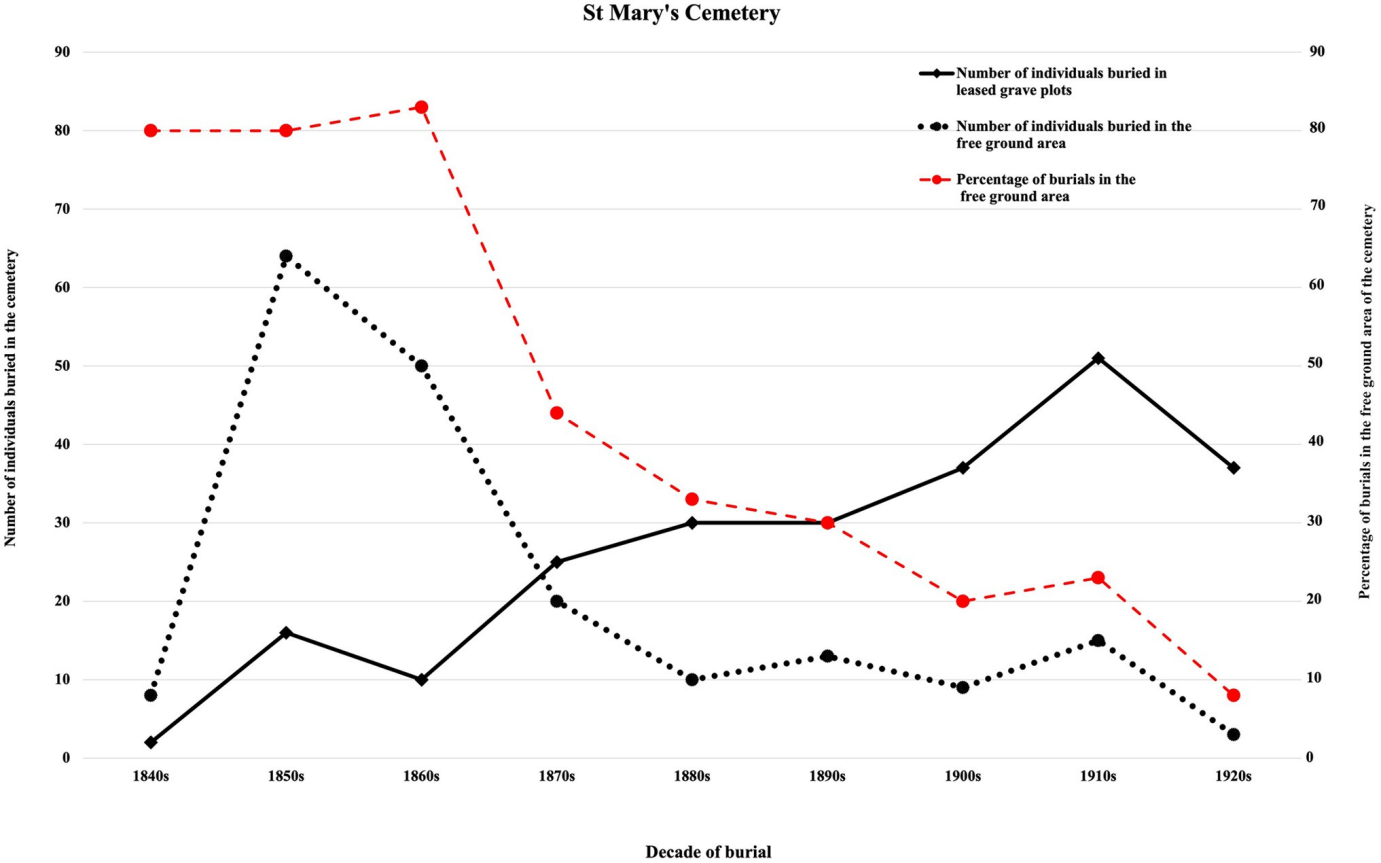
St Mary’s burial records. A comparison of the number of people listed a either buried in the free ground area or with no location of burial listed (n = 195), with those buried in leased plots with gravestone memorial markers (n = 227) in St Mary’s Cemetery, by decade of burial [73]. Red *dashed* line indicates the percentage of burials in the free ground area of this cemetery.

A survey of the gravestone memorials associated with privately funded leased burial plots in the main section of St Mary’s Cemetery (Fig 1B), indicated that at least 227 people were buried in these plots during the study period (1847–1927). A comparison of the 195 individuals that could have been buried in the free ground area with those buried in leased grave plots (n = 227) by the decade of burial is presented in Fig 2.

**Fig 3:**
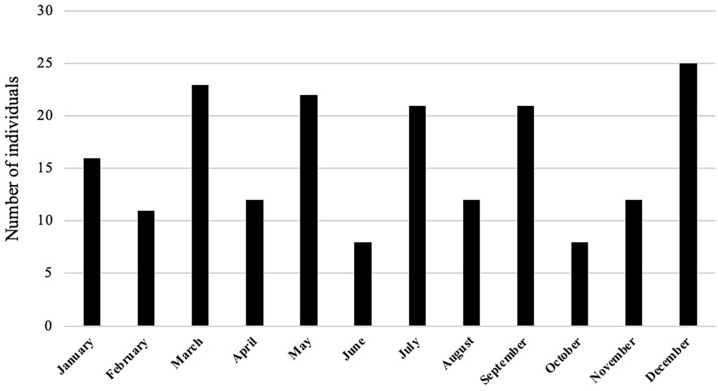
St Mary’s Cemetery—A visual representation of the number of individuals who could have been buried at the free ground with their month of burial as listed in church records (1847 to 1927) (n = 191) [73].

Analysis of individuals who could have been buried at the free ground, as listed in the church records, whose age and month of death had *also* been recorded (n = 191), over the period of 80 years (1847–1927) is presented in Table 2 and Fig 3. These findings indicated that 63% of these deaths were of infants age range 0–11 months and 64% were of subadults age range from 0–4 years (Table 2). Four individuals from the original list of N = 195 were not included in the analysis (Table 2 and Fig 3), as they either did not have their month of death recorded or their age range listed.

**Table 2:** St Mary’s Cemetery—The individuals that could have been buried at the free ground area, whose age range *and* month of death had been recorded (1847–1927) (n = 191) [73].

Month of burial:	Age range (years)										
	0–11 (months)	1–4	5–9	10–14	15–19	20–29	30–39	40–49	50–59	60+	Total
**January**	7	4	0	0	0	0	1	1	0	3	16
**February**	5	2	0	1	0	0	1	0	1	1	11
**March**	11	6	0	0	0	1	0	0	2	3	23
**April**	7	2	0	1	0	0	0	1	0	1	12
**May**	8	5	0	2	1	0	3	0	0	3	22
**June**	3	2	0	0	0	2	1	0	0	0	8
**July**	9	4	2	1	1	0	1	1	0	2	21
**August**	4	4	0	2	0	0	0	1	0	1	12
**September**	2	6	1	0	1	1	2	4	1	3	21
**October**	2	0	1	0	0	0	0	2	1	2	8
**November**	5	4	0	0	1	0	0	0	0	2	12
**December**	14	6	0	0	0	0	1	2	1	1	25
Total per age group	77	45	4	7	4	4	10	12	6	22	N = 191

**Fig 4:**
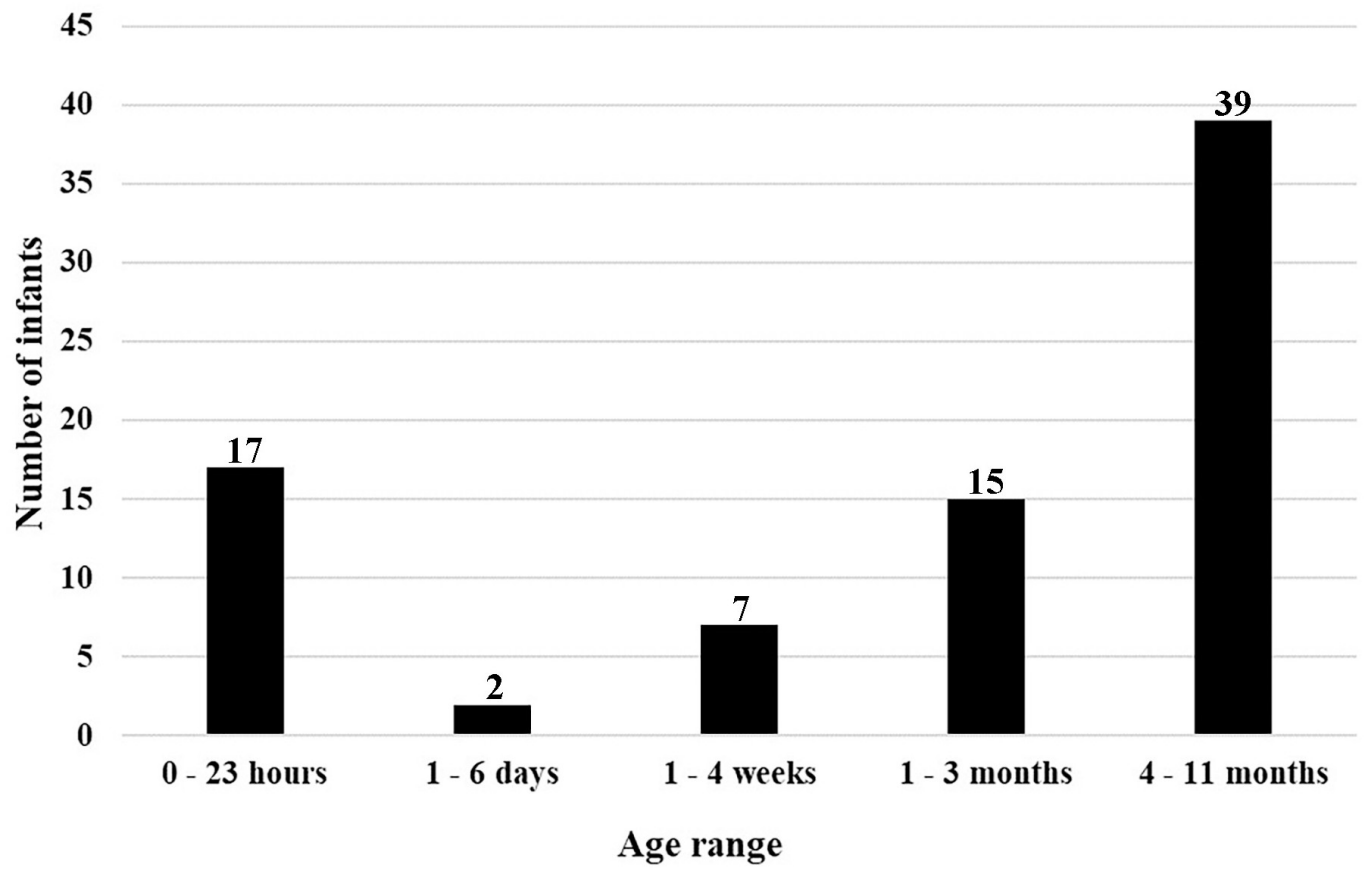
St Mary’s Cemetery—Age range at death—The number of infants under one year of age listed in St Mary’s burial records that could have been buried in the free ground area of the cemetery.

The burial records list 80 infants under the age of one year that *could* have been buried in the free ground area of St Mary’s Cemetery. As previously mentioned, there were four individuals (i.e., from the original total of N = 195) that could not be included in Table 2. Three of these four individuals were infants less than one year old, who had their *age* at death listed in the burial register but *not* the month of death. This meant that they could not be included in Table 2 or Fig 3 but they could be included in Fig 4, along with the other 77 infants already listed in Table 2 (n = 80). The fourth individual did not have either an age at death or a month of death recorded and therefore could not be included in Table 2, Figs 3 or 4.

Fig 4 represent an analysis of the ‘age range at death’ of infants less than one year of age from the burial list of N = 195 individuals that *could* have been buried in the free ground. This analysis shows that the majority of infants buried in the free ground died between 4 months and 11 months of age.

### Summary of observed skeletal manifestations

Pathological manifestations that were observed on the excavated skeletal remains of individuals buried at St Mary’s free ground area (N = 65) are presented below.

#### Bones

**Fig 5:**
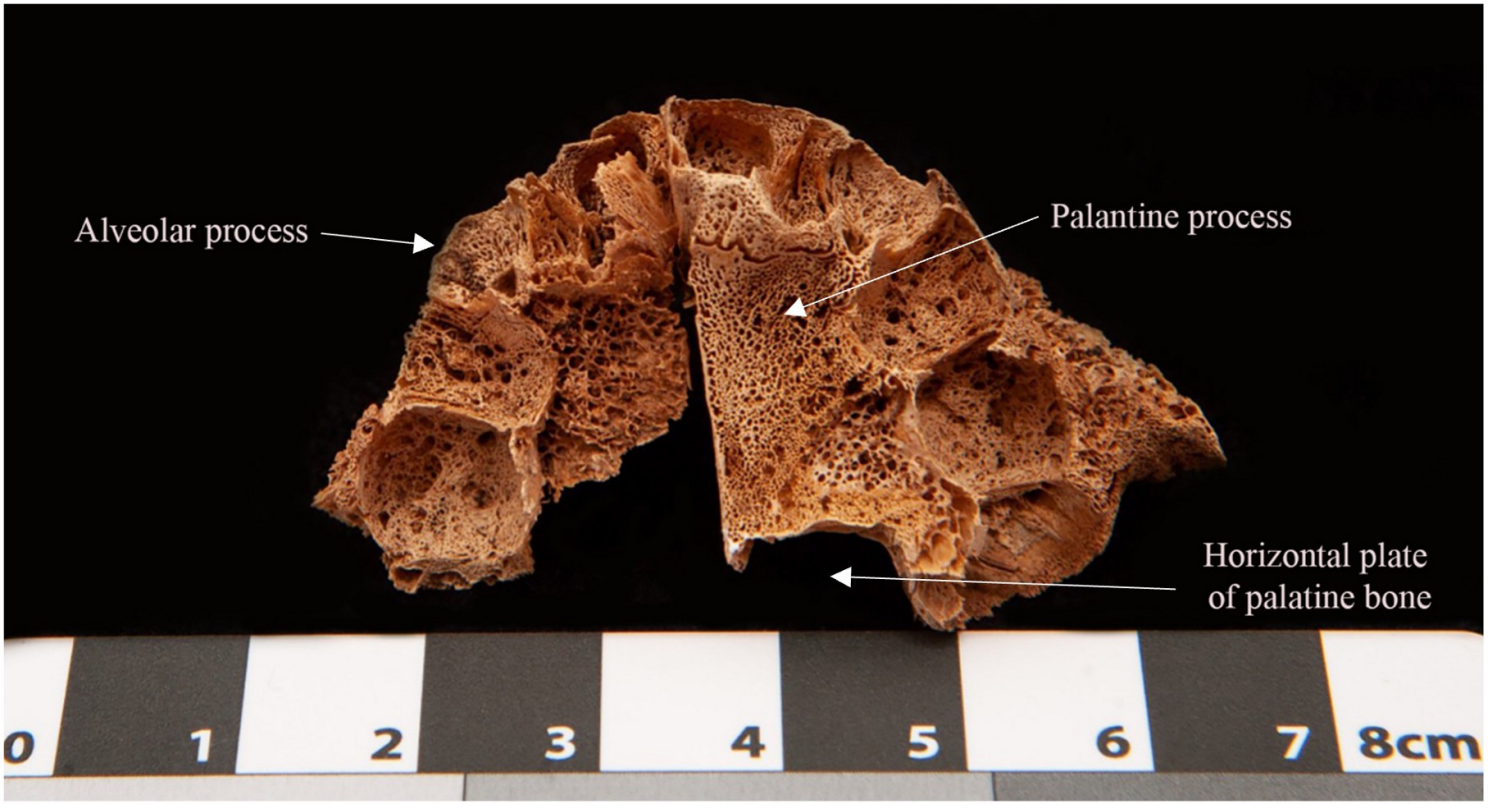
Infant, SMB 56. Palate, inferior view, showing areas of abnormal porosity that extended throughout the alveolar process of the maxillae.

**i) Abnormal porosities in the bone cortices**:

Nine adults and 12 subadults had at least one area of abnormal porosity in the cortical parts of the following bones: maxilla—infra-temporal surface, alveolar process, and palatine processes (Fig 5); mandible—medial surfaces of the coronoid process, alveolar process; sphenoid—greater wing. One infant, SMB 56 (approximately 6–9 months of age), in addition to the above bones showed areas of abnormal porosity in the cortices of the lateral and basilar portion of the occipital bone, scapulae, ribs, vertebral arches, ilia, and the extremities of long bones. The prevalences of abnormal porosities and other bony changes are presented in Table 3. The prevalences presented in the table are in relation to the number of individuals who had the particular bone.

**Table 3:** St Mary’s. prevalences of pathological manifestations associated with metabolic deficiencies in different bones of the skeletons of individuals excavated from the free ground area of the cemetery.

Age (years)	0–11 mths	1–4	5–9	10–14	15–19	20–29	30–39	40–49	50–59	60+	Total	Total Prev.%
**Abnormal porosity in the cortex of bones listed below**:												
**Maxillae**: infra -temporal surface	1/5	2/11	0/2	0/2	0/1	0/1	0/2	0/3	0/2	0/1	3/30	10
[46:215]												
Palatine proceses	1/4	0/13	0/2	0/3	0/1	0/1	0/3	1/5	1/6	0/1	3/39	8
Alveolar process	2/3	4/13	1/2	1/2	0/1	0/1	1/3	0/5	0/6	0/1	9/37	24
**Mandible**: Coronoid process medial surface	1/9	0/13	0/2	0/3	0/1	0/1	0/3	1/5	0/6	0/1	2/44	5
Alveolar process	1/9	0/15	0/2	1/3	0/1	0/1	0/3	0/5	0/7	0/2	3/48	6
Greater wing of the sphenoid bones	0/3	1/6	1/3	1/2	0/1	0/1	0/3	0/5	0/6	0/2	3/32	9
**Other skeletal changes to bones listed below**:												
**Ribs**: Enlargement of the costochondral junctions	1/4	0/5	0/1	0/2	0/1	0/1	0/3	0/3	0/2	0/0	1/22	5
**Long bones**: Flaring of the distal metaphysis	0/13	1/14	0/3	0/1	0/1	0/1	0/3	0/5	0/7	0/1	1/49	2
**Orbital roofs porous lesions** (any Type)	0/7	2/14	1/3	3/3	1/1	0/1	0/3	0/5	2/8	0/2	9/46	20
**Orbital roofs porous lesions** (Types 3 or 4) [58]	0/7	1/14	1/3	1/3	0/1	0/1	0/3	0/5	0/6	0/2	3/46	7

**Note**. Results presented as the number of individuals with the observed sign (-n-) over the total number (-N-) of individuals with the bone available for observation (n/N), with age groups of the individuals.

**ii) Enlargement and flaring of the costochondral junctions of ribs**:

One infant, SMB 56, had bilateral enlargement and flaring of the costochondral junctions of ribs (Table 3).

**iii) Enlargement and flaring of the metaphyses**:

One subadult, SMB 8 (approximately 18 months of age), showed flaring and enlargement of the distal metaphyses of the femora (Table 3).

**Fig 6:**
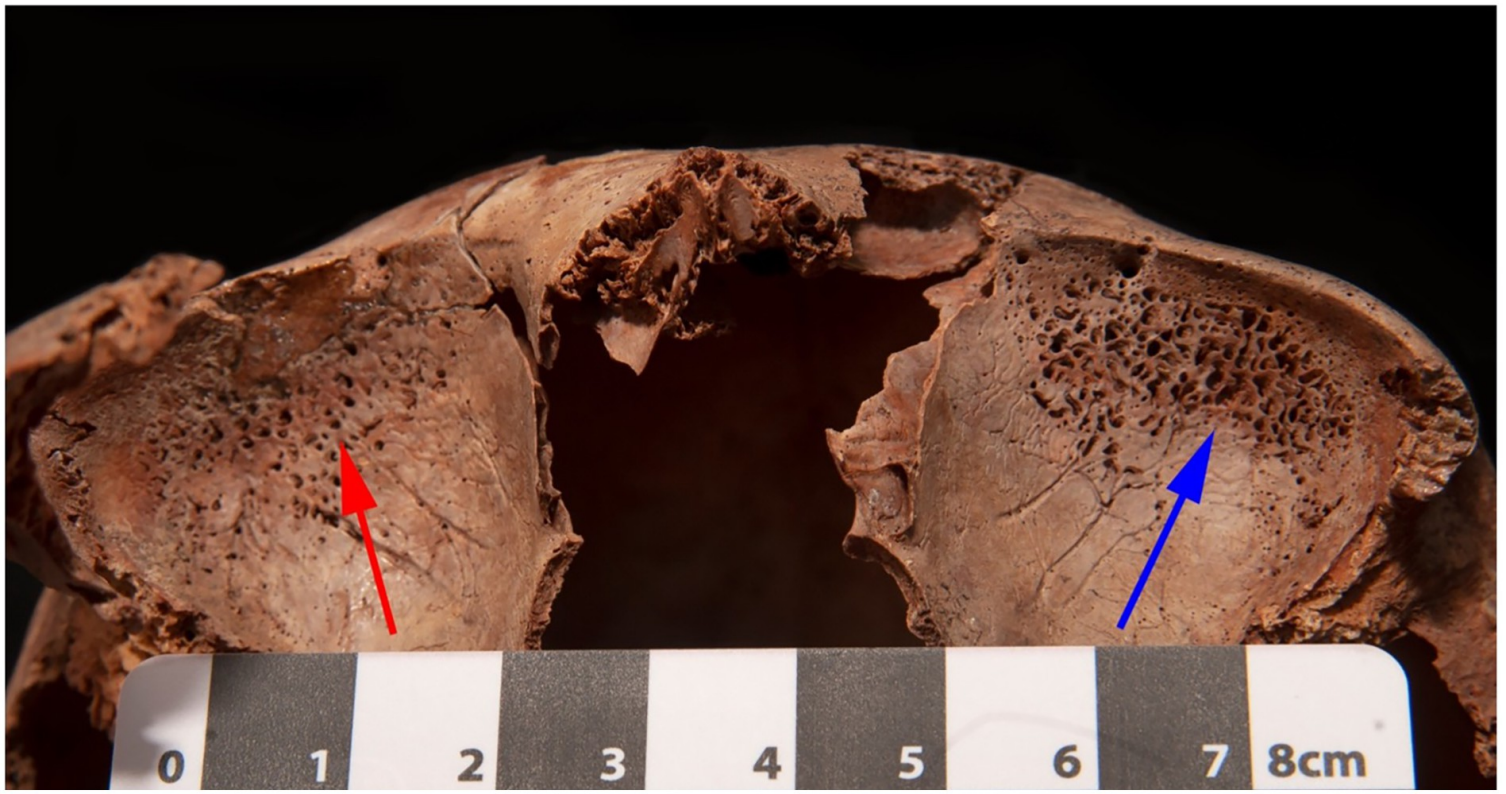
Subadult, SMB 28. Porous lesions on the bones of the orbital roof. Red arrow indicates small pores in the right bone cortex. Blue arrow indicates exposed trabecular bone on the left.

**iv) Porous lesions on the bones of the orbital roof**:

Three subadults, SMB 4A (approximately 4 years of age), SMB 19 (approximately 8 years of age), and SMB 28 (approximately 13 years of age), showed porous lesions on the bones of the orbital roof of Types 3 and 4 [59]. The lesions observed on SMB 28 were composed of small and large pores on the right and left orbital roof respectively (Fig 6). The pores on the right orbital roof penetrated the cortical bone, while those on the left exposed trabecular bone (Fig 6). This subadult also displayed areas of abnormal porosity in the cortex of the greater wing of the sphenoid bones bilaterally.

**v**) Subadults, SMB 27B (approximately 2 years of age), SMB 28, SMB 51 (approximately 11 years of age), SMB 56 and SMB 58 (approximately 2 years of age) who had two or more macroscopic pathological manifestations are summarised and presented in Table 4.

**Table 4:** St Mary’s. subadult samples with their age at death, who showed two or more macroscopic pathological manifestations associated with metabolic deficiencies.

	Approximate age at death (years)					
	SMB 27B	SMB 28	SMB 51	SMB 56	SMB 58	SMB 70
	~2	~13	~11	~6 to 9 months	~2	~ 9
**Abnormal porosity in the cortex of bones listed below**:						
**Greater wing of the sphenoid bones**	**--**	**P**	**--**	**--**	**A**	**P**
**Zygomatic bones**: internal surface	**--**	**--**	**--**	**--**	**P**	**--**
**Maxillae: infra-temporal surface** [46:215]	**A**	**A**	**--**	**P**	**P**	**A**
**Maxillae**: palatine process	**A**	**A**	**A**	**P**	**A**	**A**
**Maxillae**: alveolar process	**P**	**P**	**--**	**P**	**P**	**P**
**Mandible**: coronoid process medial surface	**A**	**A**	**A**	**P**	**A**	**--**
**Mandible**: alveolar process	**A**	**A**	**P**	**P**	**P**	**--**
**Orbital roofs**: Type of lesion [58:109]	**P** Type 1	**P** Type 4	**P** Type 1	**--**	**A**	**A**
**Scapulae: area of the supraspinous *or* infraspinous fossa**	**--**	**A**	**A**	**P**	**A**	**P**
**Pelvic bones**	**A**	**A**	**A**	**P**	**A**	**P**
**Other skeletal changes to bones listed below**:						
**Ribs**: enlargement of costochondral junctions	**--**	**A**	**A**	**P**	**A**	**--**

**Key: P** = condition present, **A** = condition absent, **--** = condition unobservable due to skeletal part missing

#### Teeth

**i) Enamel—hypoplastic defects**

A group of 42 individuals from the St Mary’s excavated sample (n = 65) had teeth available for examination. Eighteen of these individuals showed evidence of enamel hypoplastic defects and findings are presented in Table 5. The prevalence of this defect was higher among the adults (14 individuals—78% of this group) compared to subadults (4 individuals—22%) (Table 5). One subadult, SMB 70 (approximately 9 years of age), was previously diagnosed by Ioannou et al. [98] as having suffered from congenital syphilis and was treated with mercury. The infectious disease and toxic treatment given to this subadult would have affected the development of the teeth and enamel [98]. The full account of this individual’s diagnosis, skeletal lesions and analysis is published [98].

**Table 5:** St Mary’s sample. Individuals who showed enamel hypoplastic defects in permanent dentition.

St Mary’s burial code	Age range at death (years)	Sex	Total number of permanent teeth present	Total number of permanent teeth affected	Percentage of permanent teeth affected	Permanent tooth type & the number of teeth affected
**SMB 19**	5–9	U	21	7	33%	I x4, C x2, P x1
**SMB 70**	5–9	U	16	7	44%	I x3, C x2
**SMB 51**	5–9	U	25	2	8%	C x2
**SMB 28**	10–14	U	32	7	22%	I x4, C x 1, M1 x2
**SMB 79**	15–19	F	28	2	7%	C x2
**SMB 5**	20–29	F	5	2	40%	I x 2
**SMB 53C**	30–39	F	11	4	36%	I x4
**SMB 9**	40–49	M	23	8	35%	I x6, C x4
**SMB 66B**	40–49	M	17	8	47%	I x1, C x1, P x4, M2 x4
**SMB 73**	40–49	M	19	14	74%	I x7, C x4, P x2, M1 x1
**SMB 6**	40–49	M	14	1	7%	I x1
**SMB 57**	50–59	M	26	6	23%	I x3, C x3
**SMB 72**	50–59	M	29	6	21%	I x1, Cx2, Px1, M3 x2
**SMB 83**	50–59	M	15	6	40%	C x4, M2 x1, M x1
**SMB 59**	50–59	M	17	6	35%	I x5, C x1
**SMB 68**	50–59	M	18	6	33%	C x2, P x4
**SMB 23**	50–59	M	23	3	13%	I x2, C x1
**SMB 63**	60+	M	3	2	67%	I x2

Note: U = undetermined sex.

**Key**: For permanent tooth types: I = Incisor, C = Canine, P = either first or second premolar, M1 = first molar, M2 = second molar, M3 = third molar.

The incisors and canines (anterior teeth) (Table 5) were the most affected tooth type by enamel hypoplastic defects (linear and/or enamel hypoplastic pits [41]) in the St Mary’s samples.

**ii) Interglobular dentine (IGD)**

**Fig 7:**
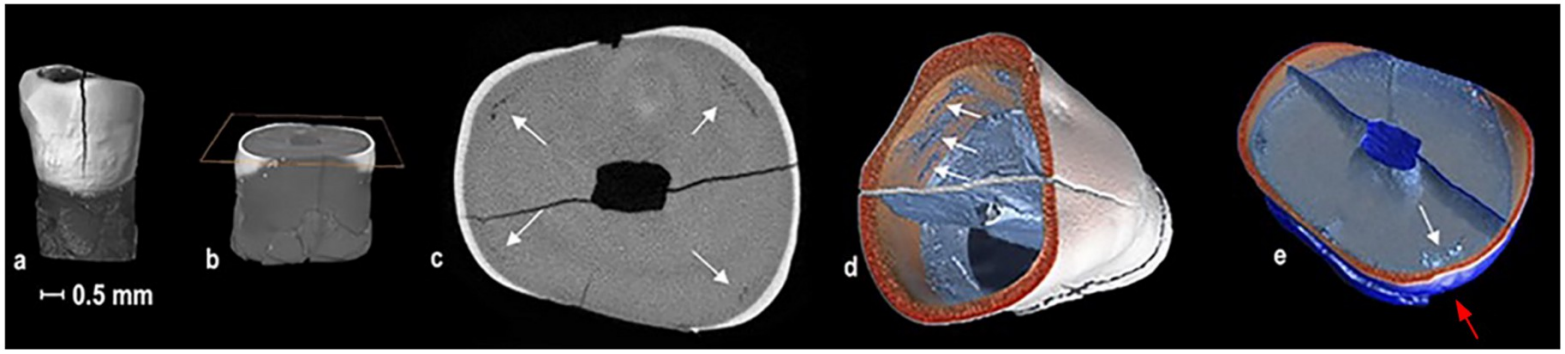
St Mary’s Cemetery. Micro-CT images. Adult, male, SMB 63. (a) Lower lateral permanent incisor with a post-mortem fracture in the crown. (b) Transverse slice: At the level of the IGD in the crown. (c) Transverse slice: Arrows show four areas of IGD. (d) Transverse slice: Arrows show three areas of IGD, image was angled to show location of IGD in three concentric layers. (e) White arrow shows IGD (internal) opposite an enamel hypoplastic defect red arrow (external).

Lesions in the internal structure of the tooth seen on micro-CT scans were areas of deficient mineralisation in the dentine. Such lesions have been described as IGD [42, 68]. Areas of IGD were seen in two adults, SMB 6 (45 to 55 years of age) and SMB 63 (55 to 60 + years of age) (Fig 7), and one subadult, SMB 70. The latter subadult was previously mentioned as the individual who had suffered from congenital syphilis and treated with mercury [98]. The tooth sample from the adult SMB 63 (a permanent lower lateral incisor) was selected or further investigation using a higher resolution of the micro-CT scanner (pixel size: 5.21 μm). This revealed areas of IGD in three separate incremental linear arrangements. One of the areas of IGD was observed opposite an external enamel hypoplastic defect (Fig 7). The crown of this tooth type commences mineralisation at approximately 30 weeks of intrauterine life (±1 month) and completes at approximately 3.5 years (±1 year) [69, 70].

### Comparison of St Mary’s findings with two British skeletal samples

Findings of the skeletal manifestations in the St Mary’s sample and the demographic profile were compared with those published for skeletal samples of two 19^th^ century British cemeteries. One sample is from St Martin’s-in-the-Bullring Church Cemetery, Birmingham, [94], and the other from St Peter’s Collegiate Church burial ground, Wolverhampton [96, 97]. A comparison of the number and percentage of adults and subadults from each sample is presented in Table 6.

**Table 6:** Demographic profiles of St Mary’s, St Martins and St Peter’s cemeteries.

Cemetery	Total Sample Size N =	Adults		Subadults	
		number	%	number	%
**St Mary’s (SA)**	65	20	31	45	69
**St Martin’s (UK)**	406	242	60	164	40
**St Peter’s (UK)**	150	92	61	58	39

### Skeletal manifestations

**Fig 8:**
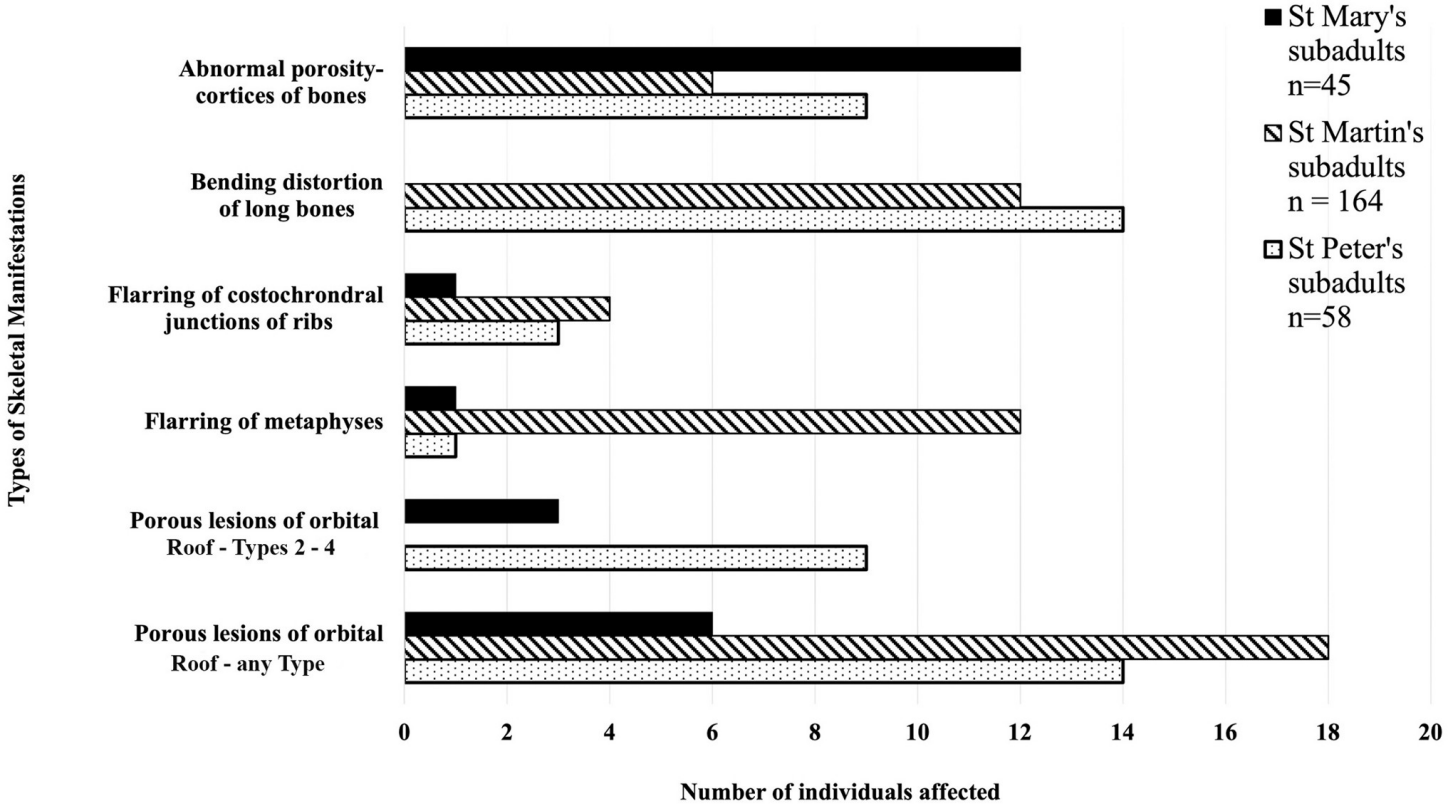
Comparison of the skeletal manifestations observed in the subadult samples from St Mary’s Cemetery, South Australia and St Martin’s and St Peter’s Cemeteries, England and the number of individuals affected.

A comparison of the macroscopically observed skeletal manifestations among the subadult samples from the three cemeteries is presented in Fig 8.

**i) Abnormal porosity in the bone cortices**:

Subadults from St Martin’s and St Peter’s Cemeteries had areas of abnormal porosity in the cortices of bones in the following anatomical sites: maxillary bone—area surrounding the infra-orbital foramen, alveolar process and the antero-medial portion of the palatine process; mandible—medial surface of the coronoid process; frontal bone—orbital plate (roof of orbit); parietal and occipital bones—external surfaces; and scapulae—supraspinous fossae [45, 94, 97]. This manifestation (abnormal porosity in the bone cortex) was higher among St Mary’s subadults compared to St Martin’s and St Peter’s subadult samples (Fig 8). Nine adults from the St Mary’s sample also had one area of abnormal porosity in the cortices of cranial bones, however, this skeletal manifestation was not observed in adults of St Martin’s or St Peter’s samples [45, 94, 97].

**ii) Bending /bowing distortion of long bones**:

This skeletal abnormality was seen only among the subadults of St Martins and St Peter’s samples (Fig 8) [94, 95, 97]. Furthermore, no adults from the three cemeteries were seen with this skeletal abnormality.

**iii) Enlargement and flaring of metaphyses**:

St Martin’s had a greater number of subadults with flaring of metaphyses than St Mary’s and St Peter’s subadult samples (Fig 8) [94, 95, 97]. No adults from the three cemeteries were seen with this skeletal manifestation.

**iv) Porous lesions on the bones of the orbital roof**:

The scores for this specific skeletal manifestation were not available for St Martin’s samples, thus could not be compared between St Mary’s and St Peter’s samples. However, the published results for St Martin’s Cemetery state that 17 adults and 18 subadults were observed with “varying degrees” of porous lesions on the bones of the orbital roof [94:135]. These results have been included in Fig 7 as porous lesions of any Type (i.e., according to the description supplied by Stuart Macadam [59:109]). Therefore, St Martin’s had the highest number of subadults with porous lesions on the bones of the orbital roof of any Type compared to St Peter’s (14 subadults) and St Mary’s (6 subadults) samples [94, 97]. A comparison of porous lesions of Types 2 to 4 [59:109] showed that St Peter’s had nine subadults with Types 2 and 3, while St Mary’s had three subadults with Types 3 and 4 (Fig 8).

## Discussion

### St Mary’s Cemetery free ground burial records

St Mary’s burial records and data collected from headstone of leased burial plots for the decades of 1840s, 1850s and the 1860s, show that the majority of people interred in St Mary’s Cemetery, during the early years of this colonial settlement, were buried in the free ground section (Fig 2). This indicates that for approximately 30 years or more, many individuals who were buried at this cemetery or members of their family could not pay for a burial. This was a period of establishment for the new colony, during which time the new settlement experienced an economic recession and had a high unemployment rate [13, 20]. Therefore, it is likely that a percentage of the early settlers in the region of St Marys-on-the-Sturt may not have had regular employment. If individuals or families had economic difficulties, they may have had to depend on charitable organisations or the government for their survival. This early economic hardship in the colony is reflected in the need for relief from the Destitute Asylum for many people living in or near the city of Adelaide.

A high mortality rate for infants is also reflected in the burial records from St Mary’s (Table 2 and Fig 4). This could be the result of many factors, including poor living conditions, lack of social/family support systems and/or inadequate availability of health services. An analysis of the month of burial, for individuals listed in the St Mary’s Church records [73], that could have been interred in the free ground area of the cemetery from 1847 to 1927, (n = 191) (Table 2 and Fig 3), showed that a higher number of infant burials took place in the summer month of December (Table 2). However, the total number of burials per month for this group (any age group) (Fig 3) showed no seasonal pattern. The weather during a South Australian summer could be extreme, with temperatures above 100ºF (38ºC). In December 1897, a local newspaper in Adelaide reported a record heat wave since the foundation of the colony, with temperatures of “over 90°F in the shade for 17 days followed by temperatures of over 100°F in the shade for a further eight days” [99:5]. They described that multiple deaths occurred in and around the city of Adelaide due to the heat, including eight deaths at the Destitute Asylum in two days [99:1]. In addition, a number of children were affected by the high summer temperatures and the public were advised that they could “be brought around by applying ice to the head” [99:1]. The findings from St Mary’s burial records and the information from this 19^th^ century newspaper suggests that the summer temperatures in South Australia could have magnified any health conditions that an individual may have been suffering. This finding contrasts with the published results for colonial settlers buried at Milton, Otago, New Zealand by Buckley et al. [100]. They were able to identify a pattern in the monthly burials at Milton, with a higher number of deaths occurring in the winter months of June, July and August and fewer deaths in the summer months [100]. The difference in seasonal trends may well be related to the different climatic conditions in South Australia and the South Island of New Zealand [100].

At St Mary’s Cemetery, the location of burial for some infants was not documented in the burial records [73:364]. This may be the result of an infant having been stillborn or dying a short time after their birth (Fig 4). The burial records listed 41 infants that were under the age of three months (Fig 4), which is 21% of the total number of individuals whose age at death was *also* listed, that could have been buried in the free ground area (n = 194—one individual did not have their age recorded). In the new colony no official regulations were in place in relation to the location of a burial for a stillborn infant, or the requirement to register the birth of a stillborn child until 1936 [74, 101]. Burial practices of perinates excavated at the 19^th^ century Parramatta Convict Hospital in New South Wales, Australia, showed they were afforded very little respect or care [102].

### Economic recovery of the new colony

The improved economic condition of the colony following the first depression during the 1840’s could have influenced the economic changes seen at St Marys-on-the-Sturt. A local financial recovery is reflected in the lower number of burials in the free ground section of St Mary’s Cemetery (Fig 2) [13, 20]. The percentage of these burials began to decrease during the 1860s and reduced to only 8% of the total burials in the cemetery in the 1920s (Fig 2). This suggests that there was a gradual improvement in the economic status of the individuals who lived in the region of St Marys-on-the-Sturt and that the majority of the excavated skeletons studied could be those of individuals who lived during the establishment of the colony. The South Australian economy improved sufficiently for the introduction of an old age pension after the turn of the 20^th^ century which reduced the need for the Destitute Asylum [30].

### Skeletal manifestations indicating metabolic disturbance

Abnormal porous lesions seen in the cortical bone of 9 adults and 12 subadults were the common manifestations present in the St Mary’s skeletal collection (Tables 3 and 4). This abnormality could be caused by a deficiency of vitamin C, which affects the synthesis of collagen in body tissues including blood vessels and bone, resulting in weak vessel walls and defective production of osteoid [45, 51, 103]. Ortner and Ericksen [47:215] have suggested that areas of abnormal porosity seen on the infra-temporal surface could be the result of an inflammatory response to “leakage from scorbutic deep temporal arteries”. They propose that abnormal porous lesions with “fine holes that are less that 1mm in diameter” are a result of localised increased vascularity seen in response to extravasated blood from weak vessels [47:212]. Different processes, including defective mineralisation in cortical bone, could appear as porous lesions in dry bone samples resulting from the decomposition of the unmineralised bone matrix [44]. The porous lesions resulting from both vitamin C and vitamin D deficiencies are similar. Therefore, it is important to differentially diagnose the lesions from normal anatomical variation, non-specific localised infections that cause an osteoblastic response, trauma, neoplastic disorders and/or genetic causes [51, 104, 105]. Furthermore, the location, distribution and quantity of lesions on the skeleton should be considered [44].

The subadult, SMB 56 (approximately 6 to 9 months of age), had abnormal cortical porous lesions in multiple locations of the skeleton (Table 4), as well as flaring of the costochondral junctions of the ribs. These skeletal manifestations could be attributed to either vitamin C or vitamin D deficiencies or a combination of both [43, 45, 95]. The rapid growth of an infant bone produces areas of new formation in the skeleton, which could appear as porosity on the cortical surface of bone. However, this change in surface texture would not penetrate the cortex of the bone in the same way as an increase of vascularity would as an inflammatory response from the body [43, 47]. The extensive and symmetrical nature of the porosity seen in the bone cortices of multiple bones of this infant is suggestive of a chronic systemic disorder that could have disturbed the metabolism near the time of death [106]. The infant’s fragmented and missing cranial bones was a limitation to a full diagnosis and could have biased the findings. It is possible that other manifestations could have been located on areas of the skeleton, for example such as porous lesions bilaterally on the greater wing of the sphenoid bones or bones of the orbital roof that were no longer present.

Five subadults, SMB 27B (approximately 2 years of age), SMB 28 (approximately 13 years of age), SMB52 (approximately 11 years of age), SMB 58 (approximately 2 years of age) and SMB 70 (approximately 9 years of age), showed two to four skeletal manifestations associated with metabolic deficiencies (Table 4). Three of these subadults had porous lesions on the bones of the orbital roof of Types 3 and 4 [59:109] (Tables 3 and 4). These types of porous lesions have been considered as an indicator of anaemia, which as previously mentioned, may have resulted from a dietary deficiency of iron and/or vitamin B12, malabsorption of iron from the gut due to chronic diseases and/or chronic blood loss resulting from gastrointestinal parasites conditions [59, 63–66]. Subadult, SMB 28 had areas of abnormal porosity on orbital roof (Type 4) and the greater wings of the sphenoid bones (bilaterally) (Table 4). This individual could have had a co-occurence of vitamin C deficiency and anaemia, as vitamin C enchances the absorption of iron from the gut [51], therefore, a deficiency of this vitamin may have aggravated any anaemia that was present in SMB 28.

The remaining adults and subadults in St Mary’s sample only showed one manifestation on one location of the skeleton, for example subadult, SMB 8 (approximately 18 months of age) had flaring of the distal metaphyses of the femora, but without any other pathological manifestations observed in their skeleton this subadult was considered as not suffering from a chronic metabolic deficiency (Table 3). It is possible that these individuals died before changes to the bone structure could occur and the state of preservation of some of the skeletal remains may also have biased the findings.

### Dental defects

**i) Enamel hypoplastic defects**

The enamel hyperplastic defects seen in the 18 individuals from the St Mary’s sample were all on permanent teeth (Table 5). This may be due to developmental timing, as the primary (deciduous) dentition is less affected by developmental enamel defects than the permanent dentition [68, 69]. The health insult/s that caused the enamel hypoplastic defect/s occurred during the development of the specific permanent tooth types (Table 5) [42, 68, 69]. The location and distribution of these enamel defects seen in the St Mary’s sample suggest they were caused by insults between birth and 4 years of age.

The 14 adults and 4 subadults who showed evidence of these enamel hypoplastic defects represented 43% of the individuals with remaining dentition (n = 42). There are 20 adults in the St Mary’s collection, two of these adults were edentulous. Fourteen of the remaining 18 adults had hypoplastic defects of the enamel (78%). This percentage is comparable to a cohort of adults (86%) with enamel hypoplastic defects who were migrants to Milton, New Zealand, during the 19^th^ century [100]. Isotopic analysis of this New Zealand cohort indicated that none of them were born locally [107], thus the health insult that caused the enamel hypoplastic defects would have occurred in their home country [100]. This is also highly likely for the adults in the St Mary’s sample. The high percentage of adults with enamel hypoplastic defects in St Mary’s sample and the sample from Milton, New Zealand, suggests that the this dental defect was common amongst the migrant settlers.

Enamel hypoplastic defects are a general, non specific indicator of disturbance and/or disease during dental development [108]. The four St Mary’s subadults with enamel hypoplastic defects (Table 5) also had other skeletal manifestations (Table 4), which have been previously discussed. The pathological conditions suffered by these individuals may have contributed to the formation of the enamel hypoplastic defects during dental development.

**ii) Interglobular dentine**

Two adults and one subadult from St Mary’s sample had areas of IGD on micro-CT scan images. Adult, SMB 63 (55 to 60 + years of age), had three separate areas of IGD (Fig 4), which indicates that he could have suffered from three separate episodes of health insults during the development of this tooth. Again, these health insults could have occurred before migration. One of the areas of IGD in the internal structure of this tooth was opposite an external enamel hypoplastic defect. It is possible that the enamel hypoplastic defect could have resulted from the same health insult that caused the IGD (Fig 4) [109–111].

### Comparison of St Mary’s free ground skeletal samples with those of St Martin’s and St Peter’s samples in Britain

The working-class background of some of the people buried at the free ground area of St Mary’s Cemetery could be similar to those published for St Martin’s and St Peter’s cemeteries in Britain [45, 94–97]. However, the demographic profile of the St Mary’s sample is somewhat different from those of the British samples. The majority of St Mary’s sample was composed of subadults (Tables 1 and 6), with 60% of subadults under the age of four years. The cause of deaths of many subadults was from gastrointestinal conditions such as dysentery and/or vomiting from contaminated water supplies, or pulmonary conditions from bacterial infection such as whooping cough [73:372–381]. Some of these deaths may have resulted from limited access to emergency medical help in the region of St Marys-on-the-Sturt. A study of mortality records from the neighbouring state of Victoria, Australia, indicated that there had been epidemics of scarlet fever and measles between 1853–1916 [112]. These infectious diseases could have spread across the border to South Australia.

The skeletal manifestation, abnormal porosity in the cortices of bones, was seen in subadults from St Mary’s, St Martin’s and St Peter’s skeletal samples [45, 94, 96]. The prevalence of such lesions was higher among the subadults from the St Mary’s sample (13%) compared to that from St Martin’s (4%) and St Peter’s samples (2%). The lower prevalence of this manifestation (probable vitamin C deficiency) among the subadults of the British samples could be due to the availability of fresh fruits and vegetables in Birmingham and Wolverhampton. The initial difficulties and delays in establishing farms and the production of food could have caused scarcity of provisions rich in vitamin C during the first decades of the development of South Australia. Subadults affected by this deficiency from the three cemeteries may have been breastfed infants [45, 94, 96, 97]. Therefore, their insufficient intake of vitamin C may have resulted from a dietary deficiency of the mother [44, 65, 113]. Elevated nitrogen isotope values (+ 1.7‰) observed in skeletal remains of some infants from the St Mary’s sample [114], relative to those of adult females in the same sample, suggested that breastmilk was a principal source of diet for infants. However, these findings do not indicate whether the child received adequate amounts of milk during breast feeding. Infants may also have been affected by the feeding and weaning practices of that period [114–117].

The number of individuals with the manifestation of enlargement and flaring of the costochondral junctions of ribs and metaphyses of long bones was higher among St Martin’s and St Peter’s subadult samples compared to those of St Mary’s samples (Fig 8) [95, 97]. The manifestation of bending distortions of long bones was only seen among subadults of St Martin’s and St Peter’s samples and not observed in any of the subadult samples from St Mary’s (Fig 8). Skeletal remains of adults from the three cemeteries were free of the above-mentioned manifestations. These skeletal abnormalities have been linked to a chronic deficiency of vitamin D [95, 97]. Among the St Mary’s subadults the lower incidence of the manifestations linked to vitamin D deficiency (Fig 7), may well be due to the abundance of sunlight (UV rays) in South Australia.

St Martin’s had the highest number of subadults with porous lesions in the bones of the orbital roof (Fig 8) [94]. The scores for these porous lesions, as described by Stuart-Macadam [59], were not available for this sample (Brickley, personal communication, 2018), which made it difficult to compare the findings from St Martin’s with those of St Mary’s and St Peter’s samples (Fig 7). A comparison of this manifestation for Types 2 to 4 [59:109], between the St Mary’s and St Peter’s subadults showed that St Peter’s had more subadults with this pathological manifestation (9 subadults) than St Mary’s (3 subadults) (Fig 8). However, St Mary’s had one subadult with a Type 4 porous lesion compared to St Peter’s subadult samples who had Type 2 or Type 3 porous lesions on the bones of their orbital roof [59:109]. This may indicate that the St Mary’s subadult, SMB 28 with the Type 4 porous lesion may have suffered a different, or more severe systemic metabolic disturbances, or may have had a co-occurance of multiple conditions that produced this manifestation.

### Conclusion

The rare skeletal sample from the free ground of St Mary’s Anglican Church Cemetery in South Australia, generated an opportunity to understand the effects of the establishment of the new colony on the health of these migrant settlers. The abnormal manifestations seen in the bones and teeth of the individuals excavated from St Mary’s reflect the health issues they experienced. Many of these issues could have been due to the marked hardships brought about by the unprepared state of the colony for the settlement of the new arrivals and the local environmental conditions. This is supported by the higher percentage of observed skeletal manifestations indicating a deficiency of vitamin C in St Mary’s sample compared with the two British skeletal samples. The people who decided to migrate to South Australia during the 19^th^ century may have arrived with hopes of prosperity through the use of natural resources. However, economic differences were seen between migrants, who had lived in the region of St Marys-on-the-Sturt, in the number of burials at the government funded free ground compared to privately funded leased burials from the mid-1840s to 1860s, in St Mary’s Cemetery. A decrease in this number of burials in the free ground area from the 1870s to 1920s, suggests a gradual improvement in the economic status of these migrants. This local improvement reflected the economic recovery seen in the rest of the colony.

## Supporting information

S1 Table Combined table of anatomical sites and descriptive features: ‘X’ indicates which metabolic deficiency the skeletal lesion may be associated with. (DOCX)

S1 Data Observed skeletal manifestation listed with location on the skeleton. St Mary’s Cemetery: Supporting Data 1.
(XLSX)

S2 Data Summary—Enamel hypoplastic defects_macroscopic examination. St Mary’s Cemetery: Supporting Data 2.
(XLSX)
