# Supplementary material for: Health effects of European colonization: An investigation of skeletal remains from 19th to early 20th century migrant settlers in South Australia
Source: PLoS One. 2022 Apr 6;17(4):e0265878. doi: 10.1371/journal.pone.0265878 (PMC8985932; doi:10.1371/journal.pone.0265878)
Supplement: S1 Table — (DOCX) [file pone.0265878.s001.docx]

| **Table S1.** Combined table of anatomical sites and descriptive features: ‘X’ indicates which metabolic deficiency the skeletal lesion may be associated with. | | | | | |
| --- | --- | --- | --- | --- | --- |
|  | | | | | |
| **Bones affected** | **Pathological manifestation** | **Vitamin C deficiency** | **Vitamin D deficiency - rickets** | **Vitamin D deficiency -**  **osteomalacia** | **Anaemia** |
| **SKULL:** | | | | | |
| Cranium-  exterior surface of bones | Abnormal porosity of cortex | X | X |  |  |
| Cranial vault | Fine pitting, diffuse porosity spread over large area |  |  | X |  |
| Cranial vault | Late closure of fontanelles |  | X |  |  |
| Basio-cranium | Invagination if severe |  |  | X |  |
| Cranial vault- internal surface of bones | Abnormal porosity of cortex  (cribra cranii) |  |  |  | X |
| Frontal bone | Slight bossing |  | X |  | X |
| Parietal bone | Slight bossing |  | X |  | X |
| Parietal bone | Porous lesion (porotic hyperostosis) |  |  |  | X |
| Parietal bone | Craniotabes |  | X |  |  |
| Occipital bone | Craniotabes |  | X |  |  |
| Occipital bone | Porous lesion (porotic hyperostosis) |  |  |  | X |
| Orbital roof | New bone growth | X |  |  |  |
| Orbital roof | Porous or hypertrophic lesion (i.e,. cribra orbitalia)  any type | X | X |  | X |
| Orbital roof | New bone growth | X |  |  |  |
| Greater wing of the sphenoid | Abnormal porosity of cortex | X |  |  |  |
| Zygomatic (lateral-orbital surface) | Abnormal porosity of cortex | X |  |  |  |
| Zygomatic bone, internal surface | Abnormal porosity of cortex | X |  |  |  |
| Maxilla – infra-temporal surface (posterior) | Abnormal porosity of cortex | X |  |  |  |
| Maxilla - alveolar process | Abnormal porosity of cortex | X |  |  |  |
| Maxilla- area surrounding  infraorbital foramen | Abnormal porosity of cortex | X |  |  |  |
| Palatine processes | Abnormal porosity of cortex | X |  |  |  |
| Mandible -coronoid process, medial surface | Abnormal porosity of cortex | X |  |  |  |
| Mandibular ramus | Deformed |  | X |  |  |
| Mandible - alveolar process | Abnormal porosity of cortex | X |  |  |  |
| **VERTEBRAE** |  |  |  |  |  |
|  | Kyphosis (severe) |  | X | X |  |
|  | Scoliosis (severe) |  | X | X |  |
| Superior & inferior surface of vertebral bodies | Biconcave depression / compression | X | X | X |  |
| Vertebral bodies | Buckling |  |  | X |  |
| **SCAPULAE:** | | | | | |
| Lateral border | Fractures |  |  | X |  |
| Scapulae: body | Exaggerated posterior curve of (when viewed from medial side) |  |  | X |  |
| Scapulae: Superior border | Buckling/ collapse of the |  |  | X |  |
| Supra-spinous fossa area -cortical bone | Abnormal porosity of cortex | X |  |  |  |
| Infra-spinous fossa area- cortical bone | Abnormal porosity of cortex | X |  |  |  |
| **STERNUM:** | | | | | |
|  | Bending |  |  | X |  |
|  | Protrusion |  | X |  |  |
| **RIBS:** | | | | | |
|  | Pseudo fractures seen as linear ridges of irregular, spiculated bone |  |  | X |  |
|  | Complete fractures |  |  | X |  |
|  | Lateral straightening |  |  | X |  |
|  | Rib angulation, pigeon chest/ Harrison’s grove |  | X |  |  |
| Costochondral junctions | Enlargement/ flaring/ swelling/ beading- ‘scorbutic rosary’ or  ‘rachitic rosary’ | X | X |  |  |
|  | Fracture adjacent to the costochondral junction. | X |  |  |  |
| Osteo-cartilaginous junction | Transverse fractures | X |  |  |  |
| **PELVIS:** | | | | | |
| All pelvic bones | Abnormal porosity of cortex | X |  |  |  |
| All pelvic bones | New bone growth | X |  |  |  |
| All pelvic bones | Subperiosteal haemorrhage (rare) | X |  |  |  |
| Superior/ inferior pubic ramus | Pseudo fractures |  |  | X |  |
| Pubis: | Anterior protrusion |  |  | X |  |
| Pubic rami | Adjacent not opposing |  |  | X |  |
| Ilia - medial aspect adjacent to greater sciatic notch: | Pseudo fracture |  |  | X |  |
| Ilia | Protrusion into pelvic inlet |  |  | X |  |
| Iliac blade | Curvature/  folding |  | X | X |  |
| Iliac crest | Fracture |  |  | X |  |
| Acetabulae | Anterior facing |  |  | X |  |
| Acetabulae | Protrusion into pelvic inlet |  | X | X |  |
| Sacrum: S3 | Extreme ventral angulation/ pelvic obstruction |  |  | X |  |
| **LONG BONES** | | | | | |
| Femoral neck | Pseudo fracture |  |  | X |  |
| Femoral neck | Coxa vara  (angulation) |  | X | X |  |
| Femoral shaft: | Antero-lateral bending |  |  | X |  |
| Epiphysis  Adult / subadult | Small calcified spurs protrude from lateral border | X |  |  |  |
| Distal metaphyses | Flaring & swelling |  | X |  |  |
| Metaphyses | Cupping deformities |  | X |  |  |
| Growth plates | Abnormal porosity of cortex |  | X |  |  |
| Growth plates | Cupping deformities |  | X |  |  |
| Diaphysis - Adult: | Fracture in diaphysis (caused by general de-ossification without new bone growth) | X |  |  |  |
| Long bones | Bending laterally (bow legged)  Genu varum |  | X |  |  |
| Long bones | Bending medially  (knock knees)  Genu valgus |  | X |  |  |
| Cortical bone Adult: | Thin cortices, (due to bone resorption & deficient periosteal new bone formation) | X |  |  |  |

**References:**

**Vitamin C Deficiency:** Diagnostic Features

- Ortner DJ, Ericksen MF. Bone changes in the human skull probably resulting from scurvy in infancy and childhood. International Journal of Osteoarchaeology. 1997;7(3):212-20. doi: 10.1002/(SICI)1099-1212(199705)7:3&lt;212: AID-OA346&gt;3.0.CO;2-5.
- Ortner DJ, Kimmerle EH, Diez M. Probable evidence of scurvy in subadults from archaeological sites in Peru. American Journal of Physical Anthropology. 1999;108(3):321. doi: 10.1002/(SICI)1096-8644(199903)108:3&lt;321: AID-AJPA7&gt;3.0.CO;2-7.
- Brickley M. The Bioarchaeology of Metabolic Bone Disease. Boston: Elsevier Academic Press; 2008.
- Brickley MB, Ives R, Mays S. The Bioarchaeology of Metabolic Bone Disease. San Diego: Elsevier Science & Technology; 2020.
- Brickley M, Ives R. Skeletal manifestations of infantile scurvy. American Journal of Physical Anthropology. 2006;129(2):163-72. doi: 10.1002/ajpa.20265.
- Snoddy AME, Buckley HR, Elliott GE, Standen VG, Arriaza BT, Halcrow SE. Macroscopic features of scurvy in human skeletal remains: A literature synthesis and diagnostic guide. American journal of physical anthropology. 2018;167(4):876. doi: 10.1002/ajpa.23699.

**Vitamin D Deficiency:** Osteomalacia Diagnostic Features

- Ives R, Brickley M. New findings in the identification of adult vitamin D deficiency osteomalacia: Results from a large-scale study. International Journal of Paleopathology. 2014;7:45-56. doi: 10.1016/j.ijpp.2014.06.004.
- Brickley M. The Bioarchaeology of Metabolic Bone Disease. Boston: Elsevier Academic Press; 2008.
- Brickley MB, Ives R, Mays S. The Bioarchaeology of Metabolic Bone Disease. San Diego: Elsevier Science & Technology; 2020.

**Vitamin D Deficiency:** Rickets Diagnostic Features

- Brickley M. The Bioarchaeology of Metabolic Bone Disease. Boston: Elsevier Academic Press; 2008.
- Brickley MB, Ives R, Mays S. The Bioarchaeology of Metabolic Bone Disease. San Diego: Elsevier Science & Technology; 2020.
- Ortner DJ, Mays S. Dry‐ bone manifestations of rickets in infancy and early childhood. International Journal of Osteoarchaeology. 1998;8(1):45-55. doi: 10.1002/(SICI)1099-1212(199801/02)8:1&lt;45: AID-OA405&gt;3.0.CO;2-D.
- Mays S, Brickley M, Ives R. Skeletal manifestations of rickets in infants and young children in a historic population from England. American Journal of Physical Anthropology. 2006;129(3):362-74. doi: 10.1002/ajpa.20292.

**Porous lesion on orbital roof bones**_ Cribra orbitalia_- Criteria & Features

- Stuart-Macadam P. Anemia in roman Britain: Poundbury camp. In: Bush H, Zvelebil M, editors. Health in Past Societies: Biocultural Interpretations of Human Skeletal Remains in Archaeological Contexts. Oxford: British Archaeological Reserach Series; 1991. p. 101-13.
- Brickley M. The Bioarchaeology of Metabolic Bone Disease. Boston: Elsevier Academic Press; 2008.
- Brickley MB, Ives R, Mays S. The Bioarchaeology of Metabolic Bone Disease. San Diego: Elsevier Science & Technology; 2020.
- Godde K, Hens S.M. An epidemiological approach to the analysis of cribra orbitalia as an indicator of health status and mortality in medieval and post-medieval London under a model of parasitic infection. American journal of physical anthropology, 2021.
